# Effects of Aqueous Dispersions of C_60_, C_70_ and Gd@C_82_ Fullerenes on Genes Involved in Oxidative Stress and Anti-Inflammatory Pathways

**DOI:** 10.3390/ijms22116130

**Published:** 2021-06-07

**Authors:** Elena V. Proskurnina, Ivan V. Mikheev, Ekaterina A. Savinova, Elizaveta S. Ershova, Natalia N. Veiko, Larisa V. Kameneva, Olga A. Dolgikh, Ivan V. Rodionov, Mikhail A. Proskurnin, Svetlana V. Kostyuk

**Affiliations:** 1 Laboratory of Molecular Biology, Research Centre for Medical Genetics, 1 Moskvorechye St, 115522 Moscow, Russia; savinova.ekaterina96@yandex.ru (E.A.S.); es-ershova@rambler.ru (E.S.E.); satelit32006@yandex.ru (N.N.V.); kamlar@med-gen.ru (L.V.K.); dolgiko@med-gen.ru (O.A.D.); svet-vk@yandex.ru (S.V.K.); 2 Department of Chemistry, Lomonosov Moscow State University, 1-3 Leninskie Gory, 119991 Moscow, Russia; mikheev.ivan@gmail.com (I.V.M.); proskurnin@gmail.com (M.A.P.); 3 Department of Normal Physiology, I.M. Sechenov First Moscow State Medical University (Sechenov University), 11-5 Mokhovaya St, 125007 Moscow, Russia; vano121099@mail.ru

**Keywords:** aqueous fullerene dispersions, pristine fullerenes, metallofullerenes, ROS homeostasis, oxidative stress, NOX4, Nrf2, PRAR-γ, heme oxygenase 1, NAD(P)H quinone dehydrogenase 1, anti-inflammatory pathways

## Abstract

Background: Fullerenes and metallofullerenes can be considered promising nanopharmaceuticals themselves and as a basis for chemical modification. As reactive oxygen species homeostasis plays a vital role in cells, the study of their effect on genes involved in oxidative stress and anti-inflammatory responses are of particular importance. Methods: Human fetal lung fibroblasts were incubated with aqueous dispersions of C_60_, C_70_, and Gd@C_82_ in concentrations of 5 nM and 1.5 µM for 1, 3, 24, and 72 h. Cell viability, intracellular ROS, NOX4, NFκB, PRAR-γ, NRF2, heme oxygenase 1, and NAD(P)H quinone dehydrogenase 1 expression have been studied. Results & conclusion: The aqueous dispersions of C_60_, C_70_, and Gd@C_82_ fullerenes are active participants in reactive oxygen species (ROS) homeostasis. Low and high concentrations of aqueous fullerene dispersions (AFD) have similar effects. C_70_ was the most inert substance, C_60_ was the most active substance. All AFDs have both “prooxidant” and “antioxidant” effects but with a different balance. Gd@C_82_ was a substance with more pronounced antioxidant and anti-inflammatory properties, while C_70_ had more pronounced “prooxidant” properties.

## 1. Introduction

Nanomaterials are being increasingly used in medicine. According to the PubMed database, there are more than 3500 reviews related to the terms “nanoparticles” or “nanomedicine” in article titles over the past five years. Fullerenes are increasingly being used in various biomedical fields owing to their unique, size-dependent functions and physicochemical properties [1,2,3]. The possibility of modifying the surface of fullerenes [4] and incorporating heteroatoms into a carbon cage (endohedral fullerenes) [5] opens up prospects for synthesizing substances with targeted biochemical properties. Thus, fullerenes and their derivatives are used as drug-delivery systems for anticancer therapy [6], antimicrobial agents [7], and antiviral agents [7]. Among metallofullerenes, gadolinium-containing endohedral fullerenes represent a new class of effective relaxation agents for magnetic resonance imaging (MRI) [8].

Fullerenes are efficient antioxidants (C_60_ is even called a “free radical sponge”) [9,10], which allows them to be considered neuroprotective [11], anti-inflammatory, and anti-ischemic [12] agents. When applied topically, C_60_ and its derivatives were used to treat cartilage degeneration, bone destruction, intervertebral disc degeneration, vertebral bone marrow disorder, and radiculopathy [13]. Antioxidant effects of water-soluble carboxyfullerene have been proved in vivo [14]. The possibility of fullerenes acting as mitochondria protonophores allows them to be considered anti-aging antioxidants [15,16]. Hydroxylated Gd@C_82_ has antineoplastic activity simultaneously with low toxicity [17,18].

Reactive oxygen species (ROS) homeostasis is a complex metabolic system, which involves many sources of ROS, the activity of which depends on the expression of the respective genes. The most important enzymes of ROS homeostasis are NADPH oxidases, respiratory chains of mitochondria and endoplasmic reticulum, peroxidases, catalase, superoxide dismutase, and others. ROS homeostasis is closely related to inflammation, carcinogenesis, apoptosis, and cell proliferation. Several proinflammatory and anti-inflammatory signaling pathways are ROS-dependent. Therefore, studying the role of fullerenes in ROS homeostasis should include research on the effect of fullerenes on signaling pathways and gene expression.

Several studies were devoted to the effect of fullerenes on genes, primarily from the point of view of toxicity. Fullerene C_60_ derivatives cause the activation of several genes in cells [19,20], and the cell response depends on the functional groups of the fullerene. Studying the gene expression in rat lungs after whole-body inhalation exposure to C_60_ fullerene revealed that few genes involved in the inflammatory response, oxidative stress, apoptosis, and metalloendopeptidase activity were upregulated at both three days and a month post-exposure. Some genes associated with the immune system process, including major histocompatibility complex-mediated immunity, were upregulated [21]. The exposure to hydroxylated fullerenes caused shifts in gene regulation involved in circadian rhythm, kinase activity, vesicular transport, and immune response [22]. A water-soluble pyrrolidinium fullerene derivative, C_60_-bis (N, N-dimethylpyrrolidinium iodide), markedly induced apoptosis of JAK2 V617F mutant-induced transformed cells through a novel mechanism, inhibiting c-Jun N-terminal kinase (JNK) activation pathway [23]. Administration of C_60_HyFn in HHcy mice significantly reduces the serum homocysteine level, neuronal apoptosis, and the expression level of the TRPM2 gene [24]. C_60_(OH)_24_ may attenuate oxidative stress-induced apoptosis via augmentation of Nrf2-regulated cellular antioxidant capacity, thus providing insights into the mechanisms of antioxidant properties of C_60_(OH)_24_ [25]. Fullerenes mediate proliferation and cardiomyogenic differentiation of adipose-derived stem cells via the modulation of MAPK pathway and cardiac protein expression [26]. Fullerenols (hydroxylated fullerenes) inhibit the crosstalk between bone marrow-derived mesenchymal stem cells and tumor cells by regulating MAPK signaling [27].

For biological applications, the surface of fullerenes should be hydrophilized by, for example, hydroxylation [28]. Fullerenols have pronounced antioxidant activity with relatively low toxicity [29]. Moreover, pristine fullerenes can form stable hydrophilic nC_60_ aggregates in water [30]. Previously, we reported on a green, scalable, and sustainable approach to preparing aqueous fullerene dispersions (AFD) of C_60_, C_70_, and Gd@C_82_ and their derivatives using sonication with an immersed ultrasonic probe [31]. Despite the apparent advantages of aqueous dispersions of pristine fullerenes, their biochemical properties have been studied exceptionally poorly. AFD stimulated the natural heterotrophic bacterioplankton and inhibited the bactericidal activity of antibiotics [32]. We have shown that AFDs of C_60_, C_70,_ and C_82_ are moderate scavengers of superoxide anion radicals [33]. We failed to find systemic studies on the effect of aqueous dispersions of pristine fullerenes and endohedral fullerenes on the oxidative stress in cells and the cell cycle.

Here, we have studied the effects of the aqueous dispersions of C_60_, C_70_, and Gd@C_82_ on ROS homeostasis in human fetal lung fibroblasts concerning: (1) cell toxicity, (2) intracellular ROS, (3) NOX4 regulation, (4) NRF2 pathway modulation, (5) NFκB pathway modulation, and (6) expression of heme oxygenase 1 and NAD(P)H quinone dehydrogenase 1.

## 2. Results

### 2.1. Preparation and Characterization of Aqueous Fullerene Dispersions

AFDs have been prepared by direct ultrasound sonication of pristine C_60_, C_70_, and Gd@C_82_ (*C_2v_*) in ultrapure water. The direct sonication procedure without solubilizing agents was previously developed to synthesize fullerene derivatives [34]. We used an immersion ultrasound probe made of titanium. Despite the formation of titania nanoparticles during ultrasonication, this procedure produces efficiently dispersed nanoparticles [35,36]. According to ICP–OES, a prolonged ultrasound exposure of fullerene C_60_–water mixtures resulted in the concentration of total titanium as low as 3.50 ± 0.05 ppm. Cellulose syringe filters with a pore diameter of 0.45 and 0.22 μm were used to purify the dispersions from titanium. As a result, all the prepared samples contained less than 1 ppm titanium dioxide.

The sizes of colloidal particles in AFDs estimated by dynamic light scattering (DLS) are in the range 90–115 nm. The DLS profiles are shown in Figure S1 in the Supplementary Materials. Fullerene clusters were negatively charged with zeta potentials about −30 mV, which characterizes AFDs as stable colloidal systems.

The polydispersity index (PDI) characterizes the mono- or polydispersity of colloidal systems. According to ISO 22412: 2008, the polydispersity index is usually less than 0.1 for monodisperse samples. For C_60_, the PDI value was 0.109, which indicates a slight agglomeration and deviation from the monodispersity. For C_70_, the PDI is 0.160, which may indicate a significant difference in the structure of the clusters. The PDI for Gd@C_82_ is between the PDI values for C_60_ and C_70_.

The details of the prepared samples are presented in Table 1.

Photographs of the aqueous fullerene dispersions are presented in Figure S2, and TEM images are presented in Figure S3 in the Supplementary Materials. The average cluster size assessed with TEM was approximately 100 nm that correlates sufficiently with the data obtained with dynamic light scattering.

**Figure 1:**
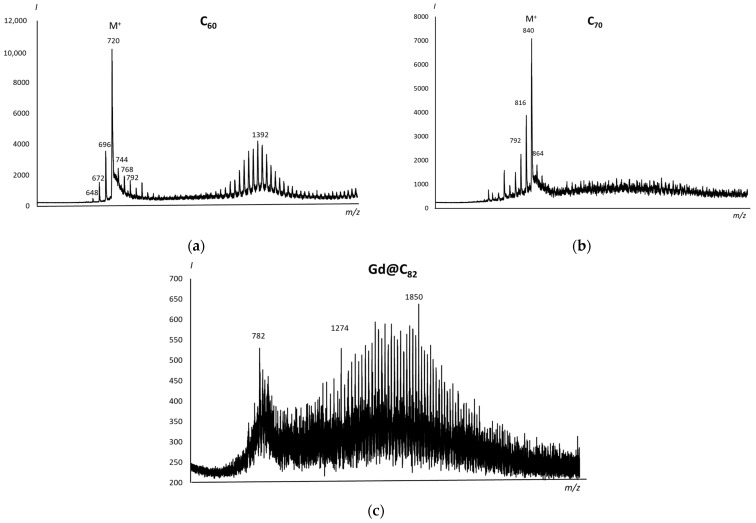
MALDI positive-ion mass spectra of AFDs of (**a**) C_60_, (**b**) (C_70_), and (**c**) Gd@C_82_ in an α-cyano-4-hydroxycinnamic acid matrix.

The purity of the obtained AFDs was checked by the MALDI method. All the spectra contain an intense molecular ion M and adducts of M with C_2_ (M + 24n) (Figure 1). The spectra show no signs of fullerene modification. The presence of adducts in the mass spectra can be explained by the coalescence phenomenon [37].

Surface functional groups were assessed by Fourier-transform infrared spectroscopy (FTIR). ETIR spectra are presented in Figure S4 in the Supplementary Materials. Main fullerene bands are present in all FTIR spectra with no bands indicating significant modifications of the fullerene surface. To improve water evaporation, we increased the diamond ATR crystal temperature up to 50 °C. For C_60_, we have observed main bands at 526, 578, 1180, and 1428 cm^−1^ which are characteristic for C_60_ [38]. There were no bands related to OH- or epoxy groups as reported in [39]. For C_70_, the main bands were as follows: 1429, 1173, 1134, 793, 723, 674, 578, 535, 457 cm^−1^ which agrees with the previously obtained data [38,40].

Due to the low IR absorption and a large number of absorption bands, we could not register a resolved FTIR spectrum for Gd@C_82_. Characteristic IR bands at 1116, 1022 and 995 cm^−1^ were not resolved and only 1063 cm^−1^ resolved band was observed [41]. Just like for C_60_ and C_70_, there were no bands related to OH- or epoxy groups.

### 2.2. Cell Viability

Cell viability was examined by a conventional MTT assay. Aqueous dispersions of C_60_, C_70_, and Gd@C_82_ were studied in a wide concentration range from 15 pM to 8.2 µM, from 15 pM to 9.1 µM, and from 0.2 pM to 105 nM, respectively. The fullerenes were incubated with cells for 72 h.

**Figure 2:**
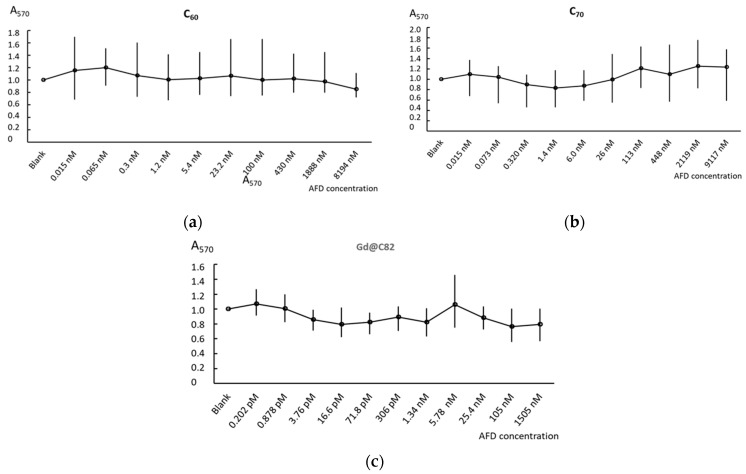
The MTT test: cell viability in versus concentrations of C_60_ (**a**), C_70_ (**b**), and Gd@C_82_ (**c**) after 72 h of incubation. In blank experiments, cells were incubated without the AFDs.

For all studied concentrations, the aqueous dispersion of C_60_ did not have toxic effects on cells and only at 8.2 µM decreased cell viability by 20% (Figure 2a). The aqueous dispersion of C_70_ decreased cell viability by less than 20% at 15 pM–1.4 nM concentrations. Interesting in concentrations of 1.4 nm–9.1 µM, C_70_ stimulated cells. Cell viability increased approx. at 20% (Figure 2b). Adding Gd@C_82_ was the least favorable for cells. Starting from 0.9 pM, this compound decreased cell viability by 20% (Figure 2c). To sum, C_60_ and C_70_ AFDs were non-toxic in concentrations less than 9 µM and Gd@C_82_ was non-toxic in concentrations less than 0.1 µM.

### 2.3. Mitochondrial Potential

**Figure 3:**
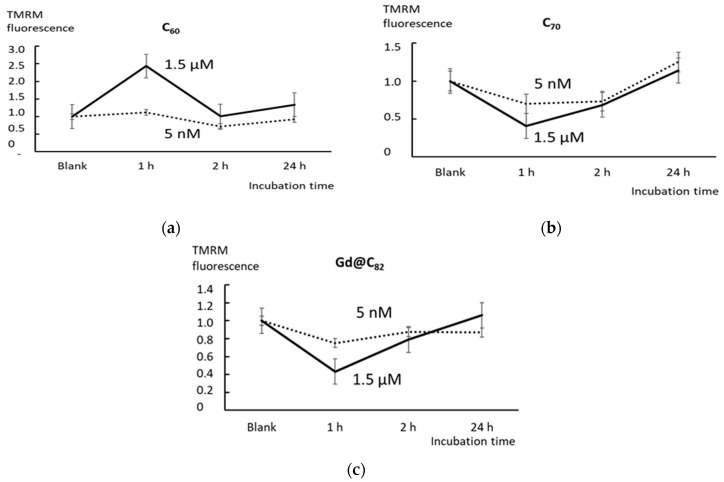
The mitotracker test with TMRM using flow cytometry: the relative enhancement of the mitotracker signal in cells relative to the blank versus incubation time with C_60_ (**a**), C_70_ (**b**), and Gd@C_82_ (**c**). In blank experiments, cells were incubated without AFDs. Concentrations are indicated in the figure.

The MTT test is commonly used to assess cellular metabolic activity, mainly to assess mitochondrial functionality. Next, we analyzed the response of HFLF mitochondria to the addition of AFDs. Using flow cytometry, we found that the intensity of the TMRM signal in the mitochondria increased by a factor of 2.4 after 1 h of incubation with 1.5 μM C_60_. After 3 h of incubation, the signal level returned to the blank values. After 24 h of incubation, we observed a slight increase in the signal level. In concentrations of 5 nM, C_60_ did not significantly affect the mitochondrial potential (Figure 3a).

Fullerene C_70_ (both 5 nM and 1.5 μM) and Gd@C_82_ (1.5 μM) after 1 h of incubation decreased the mitochondrial potential. After 3 h of incubation, the mitochondrial potential increased. After 24 h of incubation, the mitochondrial potential reached blank values. Cd@C_82_ at a concentration of 5 nM did not have a statistically significant effect on the mitochondrial potential (Figure 3b,c).

Decreasing the TMRM signal in cells can be caused by two processes. The first cause is quenching the mitotracker by fullerene; the second is capturing energy by C_70_ and Gd@C_82_ from the TMRM dye. These processes can occur given the close contact of fullerene molecules with a mitotracker provided in mitochondria. This conclusion is confirmed by several studies on the localization of fullerenes in mitochondria [42,43].

### 2.4. Localization of Fullerenes inside Human Fetal Lung Fibroblasts

**Figure 4:**
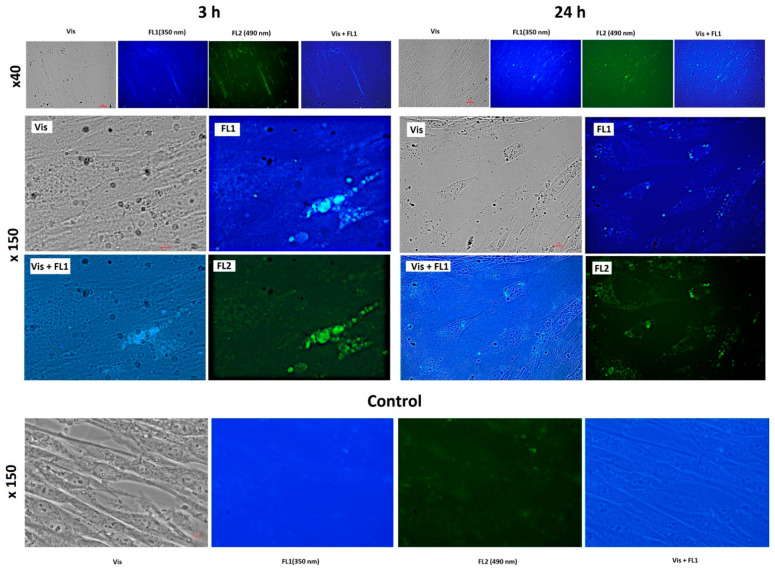
Fluorescence of C_60_ (concentration 1.5 µM) in the cells after 3 and 24 h of incubation: Vis, transmitted light, fluorescence was registered with a 450–525 nm filter, excitation wavelength 350 nm (FL1) and 490 (FL2); magnification is shown at the left. Scale is marked with red bars. The bottom series of photographs are images of cells incubated without the fullerene (a control experiment).

**Figure 5:**
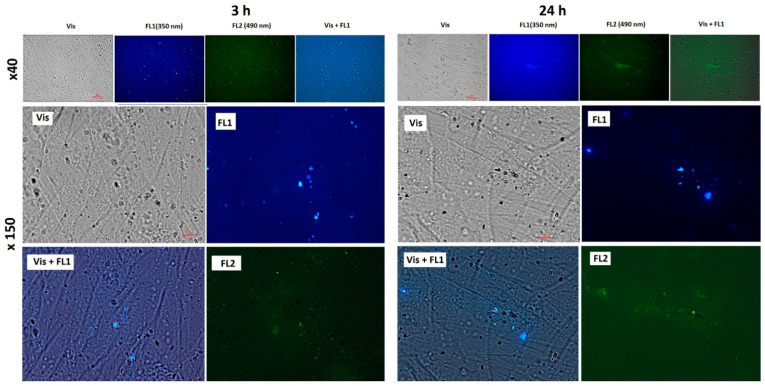
Fluorescence of C_72_ (concentration 1.5 µM) in the cells after 3 and 24 h of incubation: Vis, transmitted light, fluorescence was registered with a 450–525 nm filter, excitation wavelength 350 nm (FL1) and 490 (FL2); magnification is shown at the left. Scale is marked with red bars.

**Figure 6:**
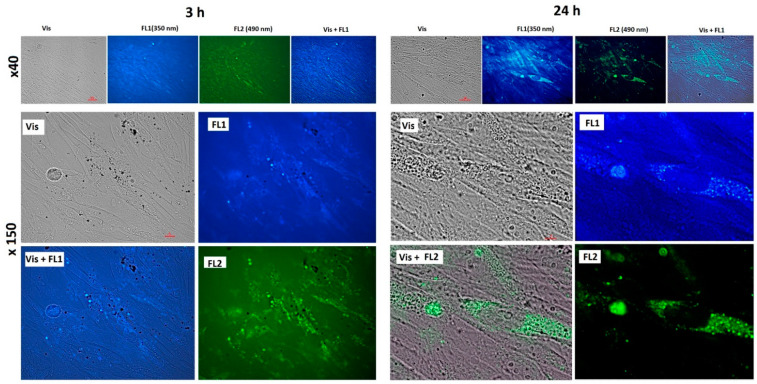
Fluorescence of Gd@C_82_ (concentration 1.5 µM) in the cells after 3 and 24 h of incubation: Vis—transmitted light, fluorescence was registered with a 450–525 nm filter, excitation wavelength 350 nm (FL1) and 490 (FL2); magnification is shown at the left. Scale is marked with red bars.

To study the penetration and distribution of the fullerenes in cells, fluorescence imaging was registered. Fluorescence images of cells incubated for 3 and 24 h with C_60_, C_70_, and Gd@C_82_ are shown in Figure 4, Figure 5 and Figure 6, respectively.

The photographs show that after 3 h of incubation, the fullerenes are localized mainly at the cell surface. The absorption of fullerenes was not strong as they were washed off during washing. After 24 h, fullerenes penetrate the cell membrane into the cytoplasm. The photographs clearly show dark rounded nuclei, into which fullerenes do not penetrate. The results of flow cytometry confirm the data of fluorescence microscopy.

### 2.5. Intracellular ROS

After incubation with AFDs, intracellular ROS were detected with H2DCFH-DA (2,7- dichlorodihydrofluorescein diacetate) using a flow cytometer. After permeation into a cell, the compound is deacetylated with intracellular esterases. Free radicals oxidize the nonfluorescent H2DCFH in the cytoplasm to the highly fluorescent 2′,7′-dichlorofluorescein. Figure 4 shows the histograms of DCF production in the cells after adding C_60_, C_70_, and Gd@C_82_ related to blank cells versus the incubation time.

**Figure 7:**
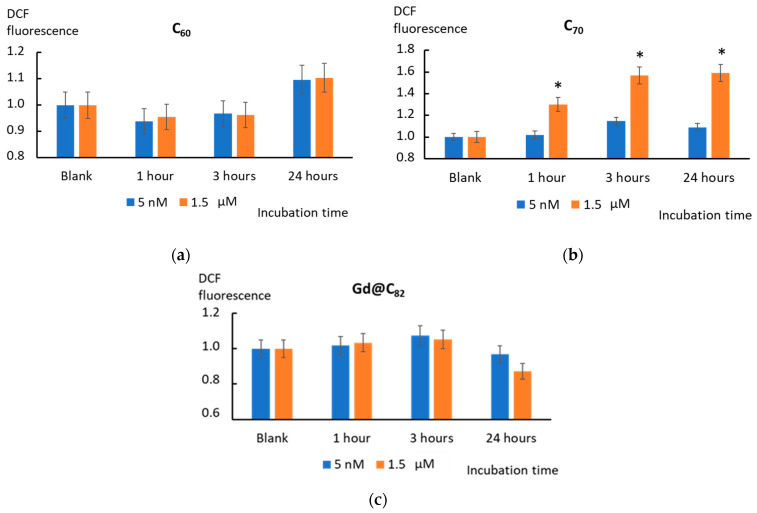
The histograms of intracellular ROS production on the incubation time for C_60_ (**a**), C_70_ (**b**), and Gd@C_82_ (**c**) at the concentrations of 5 nM and 1.5 µM; flow cytometry was used. (*) denotes significant differences with the blank cells, *p* < 0.01, a nonparametric U-test.

C_60_ added to the cells tends to decrease the ROS level in cells after 1 and 3 h regardless of the concentration. Intracellular ROS is lowered despite the high activity of the NOX4 enzyme (see below, Section 2.6) and mitochondria, which promote ROS synthesis. After 24 h of incubation, the ROS level increased above the control values (Figure 7a).

Incubation with C_70_ (1.5 µM) caused a significant increase in the ROS level (*p* <0.01) after 1, 3, and 24 h incubations. At a nanomolar concentration (5 nM), the ROS level did not increase significantly (Figure 7b).

Incubation with Gd@C_82_ resulted in a slightly increased ROS level after 3 h of incubation. After 24 h, a decrease in the ROS level was observed, specially marked for micromolar concentrations (Figure 7c).

### 2.6. NOX4 Expression

One of the primary sources of ROS in cells is NADPH oxidases (NOX), which are predominantly localized in endosomes [44]. We studied the expression of the NOX4 gene and protein in the cells following their incubation with AFDs of C_60_, C_70_, and Gd@C_82_.

**Figure 8:**
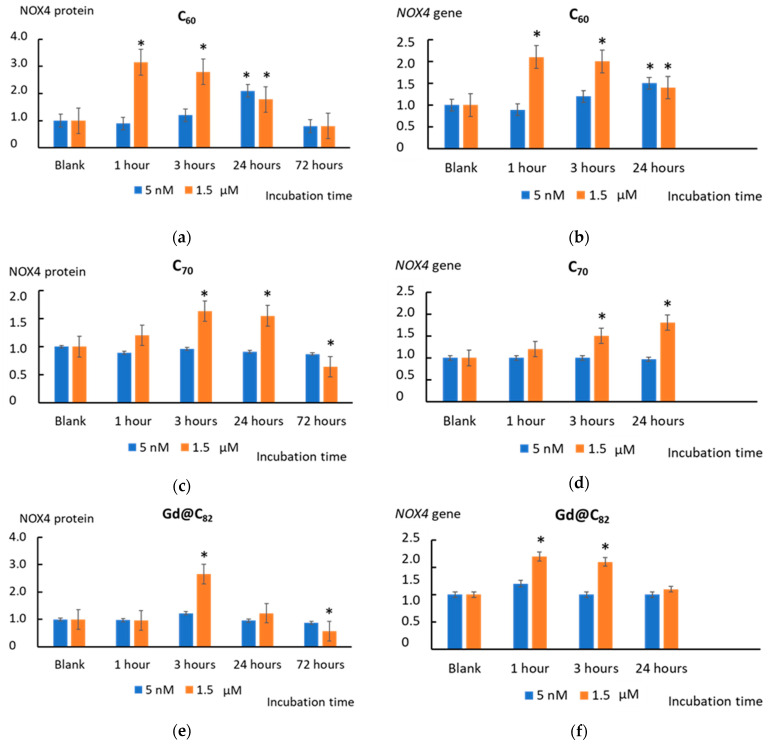
The NOX4 protein level in cells treated with (**a**) C_60_, (**c**) C_70_, and (**e**) Gd@C_82_ at the concentrations of 5 nM and 1.5 µM. The expression of the NOX4 gene in cells incubated with (**b**) C_60_, (**d**) C_70_, and (**f**) Gd@C_82_ at the concentration of 5 nM and 1.5 µM. The *NOX4* RNA level is the mean from three replicates related to the *NOX4* gene expression in the blank cells. The *TBP* gene was used as an internal-standard gene. The blank cells were incubated without the AFDs. (*) denotes significant differences with the blank cells, *p* < 0.01, a nonparametric U-test.

For 1.5 µM C_60_, the NOX4 protein level increased by three (*p* < 0.01) after 1 h of incubation and remained increased by a factor of 1.5–2.8 within 3–24 h. After 72 h of incubation, NOX4 protein decreased to a lower value than the blank. At a concentration of 5 nM, C_60_ caused an increase in NOX4 after 24 h of incubation by a factor of two (*p* < 0.01). The NOX 4 protein decreased below the blank after 72 h of incubation (Figure 8a). For 1.5 µM C_70_, the NOX4 protein level increased by a factor of 1.6–1.4 (*p* < 0.01) within 3–24 h and decreased to a value lower than the blank by 40% (*p* < 0.01) after 72 h of incubation. At a concentration of 5 nM, C_70_ did not cause significant changes in NOX4 protein expression within 72 h of incubation (Figure 8c). Fullerene Gd@C_82_ only at a concentration of 1.5 μM caused an increase in the NOX4 level after 24 h of incubation by a factor of 2.5 (*p* < 0.01) and an almost twofold decrease (*p* < 0.01) below the blank values after 72 h (Figure 8d).

NOX4 expression is regulated by its transcription. The NOX4 protein level and *NOX4* gene transcriptional activity changes agreed. The *NOX4* transcription caused by the studied fullerenes changed synchronously with the change in the protein expression level, but slightly ahead of time (Figure 8b,d,f).

### 2.7. NFκB Pathway and PRAR-γ

We have not found effects of C_60_, C_70_, and Gd@C_82_ on the NFκB pathway either for the expression of *NFKB1* gene or for the NFκB transcription factor. We also have not found any effects on the expression of target genes of NFκB pathway such as *TNFA, IL-6,* and *IL-1B.*

**Figure 9:**
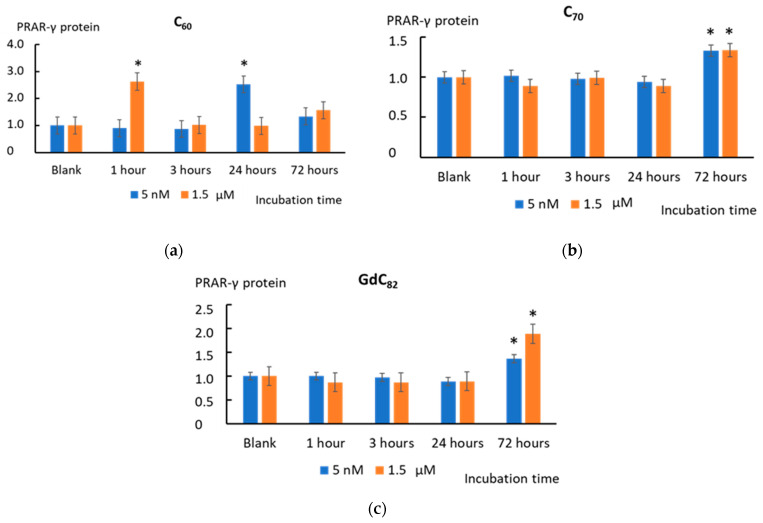
The PRAR-γ protein level in cells treated with (**a**) C_60_, (**b**) C_70_, and (**c**) Gd@C_82_ at concentrations of 5 nM and 1.5 µM. The blank cells were incubated without the AFDs. (*) denotes significant differences with the blank cells, *p* < 0.01, a nonparametric U-test.

The peroxisome proliferator-activated receptor-γ (PPAR-γ) is involved in the antioxidant response. It inhibits the NFκB signaling pathway, which leads to a decrease in the expression of proinflammatory mediator genes [45,46]. C_60_ (1.5 µM) increased PPAR-γ expression after 24 h of incubation. C_60_ (5 nM) increased the PPAR-γ expression after 1 h of incubation by a factor of 2.5 (*p* < 0.01) (Figure 9a). Fullerenes C_70_ and Gd@C_82_ caused an increase in PPAR-γ expression by a factor of 1.4–1.8 after 72 h of incubation (*p* < 0.01) (Figure 9b,c).

### 2.8. NRF2 Pathway

Cellular ROS are regulated by ROS production systems and antioxidant response systems such as the transcription factor NRF2, a component of the anti-inflammatory pathway.

**Figure 10:**
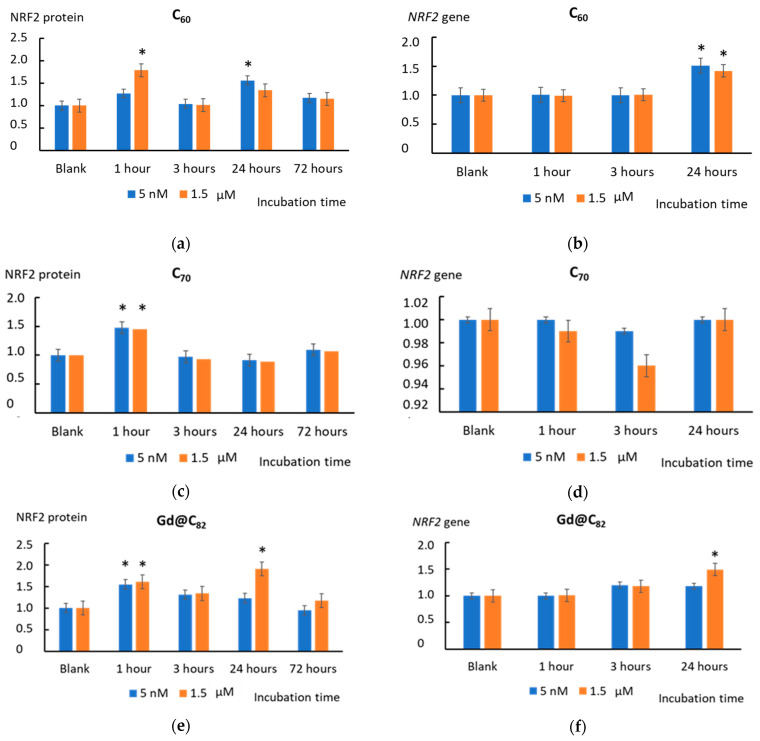
The NRF2 protein level in the cells treated with (**a**) C_60_, (**c**) C_70_, and (**e**) Gd@C_82_ at concentrations of 5 nM and 1.5 µM related to the blank cells incubated without the AFDs. The expression of the *NRF2* gene in cells incubated with (**b**) C_60_, (**d**) C_70_, and (**f**) Gd@C_82_ at concentrations of 5 nM and 1.5 µM. The *NRF2* RNA level is the mean from three replicates related to the *NRF2* gene expression in the blank cells. The *TBP* gene was used as an internal-standard gene. (*) denotes significant differences with the blank cells, *p* < 0.01, a nonparametric U-test.

The expression of NRF2 protein increased by a factor of 1.5–1.8 after 1 h of incubation with all the studied AFDs both in nanomolar and micromolar concentrations (except for 5 nM C_60_). After 3 h of incubation with C_60_ and C_70_, the NRF2 expression did not change. AFD Gd@C_82_ slightly increased the NRF2 expression (Figure 10). After 24 h, the NRF2 expression in the cells incubated with C_60_ for both concentrations increased by 30–50% and gradually decreased in 72 h (Figure 10a). AFD C_70_ did not affect the NRF2 expression after 24 or 72 h (Figure 10c). After 24 h, Gd@C_82_ in micromolar concentration caused an increase in the NRF2 level by a factor of 1.8 (*p* < 0.01). After 72 h of incubation, the NRF2 expression decreased to the blank values (Figure 10e).

After 1 h of incubation, the expression of the *NRF2* gene did not increase while NRF2 protein increased. That fact indicates the activation of NRF2 that already existed in the cells. After 24 h of incubation, the expression of the *NRF2* gene increased due to incubation with C_60_ in both concentrations and Gd@C_82_ at 1.5 µM, which leads to an increase in the transcriptional activity of the *NRF2* gene in cells (Figure 10b,d,f).

### 2.9. Heme Oxygenase 1 and NAD(P)H Quinone Dehydrogenase 1

**Figure 11:**
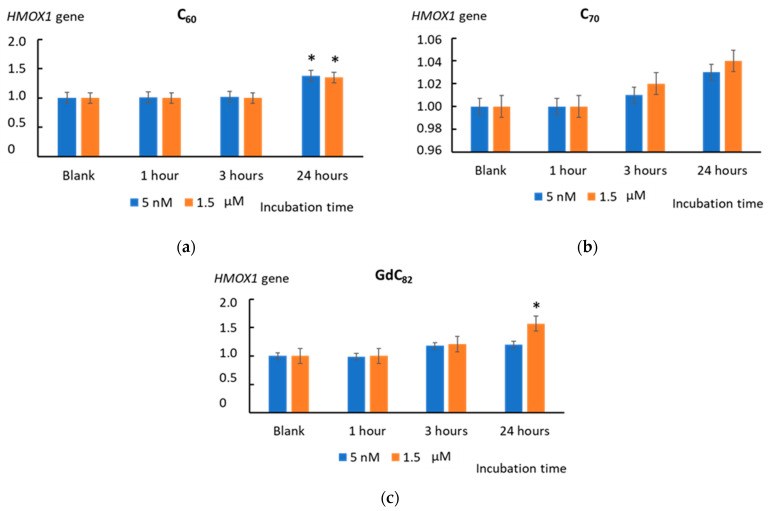
The expression of the *HMOX1* gene in cells incubated with (**a**) C_60_, (**b**) C_70_, and (**c**) Gd@C_82_ at concentrations of 5 nM and 1.5 µM. The *HMOX1* RNA level is the mean from three replicates related to the *HMOX1* gene expression in the blank cells. The *TBP* gene was used as an internal-standard gene. (*) denotes significant differences with the blank cells, *p* < 0.01, a nonparametric U-test.

We studied the transcriptional activity of the *HO-1* gene (*HMOX1*), which encodes heme oxygenase 1 and is the target gene of the transcription factor NRF2. An increase in the expression of the *HMOX1* gene was found after 24 h of incubation with C_60_ (5 nM and 1.5 µM) and Gd@C_82_ (1.5 µM) (*p* < 0.01). This fact is also evidence of the transcriptional activity of the *NRF2* gene in the cells (Figure 11).

**Figure 12:**
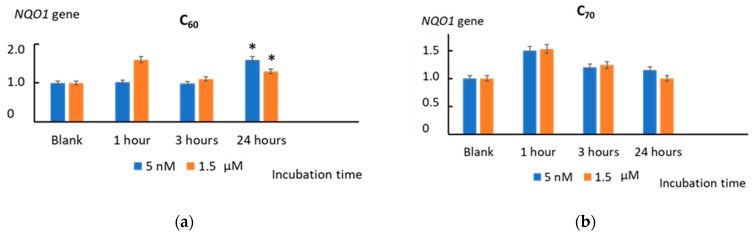

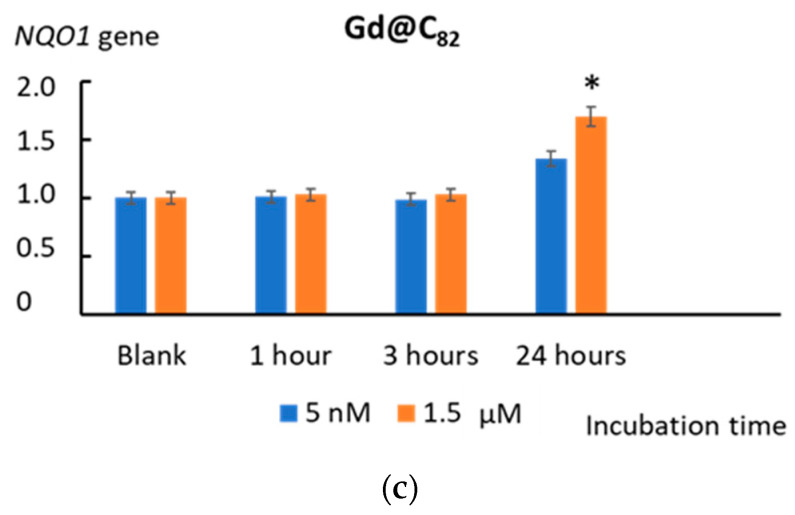
The expression of the *NQO1* gene in cells incubated with (**a**) C_60_, (**b**) C_70_, and (**c**) Gd@C_82_ at concentrations of 5 nM and 1.5 µM. The *NQO1* RNA level is the mean from three replicates related to the *NQO1* gene expression in the blank cells. The *TBP* gene was used as an internal-standard gene. (*) denotes significant differences with the blank cells, *p* < 0.01, a nonparametric U-test.

The NAD(P)H quinone oxidoreductase 1 (*NQO1*) gene is also a target gene for the NRF2 transcription factor. We found an increase in the expression of the *NQO1* gene after 24 h of incubation with C_60_ (5 nM and 1.5 µM) (*p* < 0.01) and after 24 h of incubation with Gd@C_82_ (1.5 µM) (*p* < 0.01), which also confirms the activation of NRF2 in the cells (Figure 12).

## 3. Discussion

**Figure 13:**
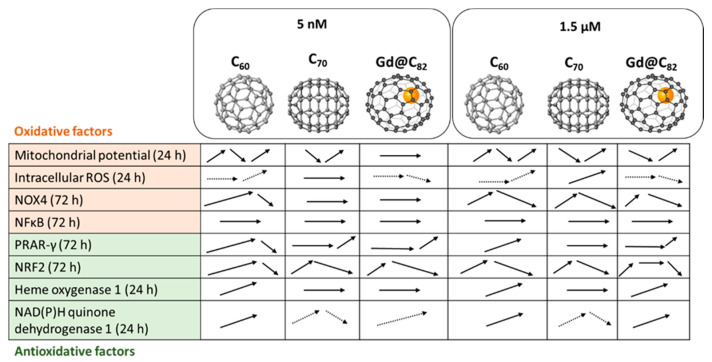
The dynamics of changes in the studied parameters over time (the time range is indicated in the first column) for two concentrations of aqueous fullerene dispersions. Solid lines denote significant changes and dashed lines, trends.

We incubated human fetal lung fibroblasts with aqueous fullerene dispersions C_60_, C_70_, and Gd@C_82_ for 1, 2, and 24 h and investigated the expression of genes and proteins of oxidative stress and anti-inflammatory response. We studied two concentrations in the nanomolar and micromolar ranges since it is known that the regulation of ROS homeostasis depends on the concentration of substances. The main results are summarized in Figure 13.

We studied two somewhat different concentrations to assess whether the effect of fullerenes on genes is monotonic or if there is a switching point. It is known that ROS-active substances exhibit pro- or antioxidant features depending on their concentration [47]. All the studied aqueous fullerene dispersions were safe for cells in a wide range of concentrations. AFDs of C_60_ and C_70_ did not cause cell death up to a concentration of 10 µM; however, metalofullerene Gd@C_82_ was less safe and caused the death of 20% cells at 100-fold lower concentrations (0.1 µM). We have selected the same concentrations for all AFDs to compare their effects. Therefore, we studied 1.5 µM, the safe upper concentration for Gd@C_82_. A concentration of 5 nM was selected because this concentration still affects the genes and proteins.

The MTT test is commonly used to assess the metabolic activity of cells, mainly to mitochondrial functionality. Therefore, we studied the effects of AFDs on the mitochondria of HFLF cells using flow cytometry. Tetramethylrhodamine, methyl ester (TMRM), is a cell-permeant, cationic fluorescent dye that is readily sequestered by active mitochondria with intact membrane potentials. This dye is a lipophilic cation accumulated by mitochondria in proportion to ΔΨ [48,49]. The mitochondrial membrane potential is a global indicator of mitochondrial function and the metabolic state of cells. Upon loss of the mitochondrial membrane potential, TMRM accumulation will cease, and the signal will dim or disappear.

Both concentrations of C_60_ and C_70_ showed similar dynamics. For the first hour, C_60_ activated mitochondria; then, their metabolism returned quickly to the initial level. A higher concentration of C_60_ had a more significant effect. For C_70_, on the contrary, the metabolism was suppressed, then returned and increased by the end of the 24-h period. The metalofullerene acted similarly in a high concentration but did not affect a low concentration. The mechanism of activation of metabolism by fullerenes is challenging to explain without considering the spatial distribution of nanoparticles in the cell, which requires further microscopic experiments.

Mitochondrial respiratory chains are the primary intracellular sources of ROS because, in oxidative phosphorylation, superoxide anion radicals leak from the chain. After 24 h of incubation, C_60_ and C_70_ caused an increase in the activity of mitochondria. Thus, they can be considered mitochondrial “prooxidants”.

We have previously shown that concerning the superoxide anion radical fullerenes can be arranged in the row Gd@C_82_ > C_60_ > C_70_ for the ability to scavenge SAR; and C_60_ and C_70_ differ in the mechanism of interaction with SAR from SOD, which allows them to be instead considered superoxide scavengers, in contrast to Gd@C_82_, which, presumably, is a SOD mimic [33]. This result is consistent with the electron affinity row C_60_ < C_70_ < Gd@C_82_. The insertion of Gd into a C_82_ cage increases the electron affinity to 3.3 eV [50]. Moreover, the high polarizability of fullerenes facilitates the attachment of radicals to their surface [51].

As for intracellular ROS, C_60_ and C_70_ showed a similar trend (Figure 7a,b). After 24 h of incubation, ROS increased. However, these changes were significant for a high concentration of C_70_, possibly because C_60_ is a stronger antioxidant for the superoxide anion radical. Metallofullerene is an even stronger antioxidant than C_60_. It also did not have an activating effect on mitochondria. Perhaps that is why, after incubation with Gd@C_82_, intracellular ROS decreased.

Besides mitochondrial electron transport chains, NADPH oxidases are critical sources of ROS. Trends in protein and gene expression were similar. Both concentrations of C_60_ caused an increase in NOX4 expression. The effect of C_70_ and Gd@C_82_ was achieved for a higher concentration. Therefore, C_60_ is a more active stimulus for the NOX4 system.

The studied fullerenes do not affect the NFκB, but they do affect the expression of PPAR-γ, which is sensitive to oxidized lipids [52]. C_70_ and Gd@C_82_ had a pronounced effect after 72 h of incubation, and C_60_ acted much more actively in the first hours. We have previously found that C_60_ is a very weak inducer of lipid peroxidation (unpublished data), while C_70_ and Gd@C_82_ at the studied concentrations did not induce lipid peroxidation. This behavior may explain the rapid increase in the expression of this gene in response to incubation with C_60_.

Summing up the “prooxidant part,” all studied fullerenes directly affected mitochondrial metabolism and activated NOX4, with C_60_ having the most significant effect. Perhaps this is due to its smaller molecule size. The incubation with fullerenes led to an increase in intracellular ROS, except for Gd@C_82_. Perhaps this is due to the balance of its pro- and antioxidant properties. Gd@C_82_ is a weaker prooxidant and a stronger antioxidant concerning the superoxide radical. Moreover, it exhibits SOD-like properties [33].

The development of intracellular oxidative stress leads to the activation of the NRF2 anti-inflammatory pathway. NRF2 in normal cells in the complex with the cytoskeleton protein KEAP1 is inactive. This complex lies mostly in a cytoplasm [53,54,55]. After modifying KEAP with ROS, NRF2 dissociates from KEAP and moves into the nucleus. In the nucleus, NRF2 reacts with ARE (antioxidant response elements) of genes, coding the enzymes for detoxication and cytoprotective proteins such as NAD(P)H quinone dehydrogenase 1 (NQO1) and heme oxygenase 1 (HO-1) [56].

The incubation of cells with all AFDs activated this pathway within one hour. C_60_ and Gd@C_82_ had a long-term effect of up to 24 h. The effect of C_70_ was expressed within one hour, and no gene expression was found. An increase in the transcriptional activity of the NRF2 gene in cells is also evidenced by an increase in the transcriptional activity of the HMOX1 and NQO1 genes, which are the target genes of the NRF2 transcription factor. A significant increase in expression was obtained for C_60_ and Gd@C_82_, but not for C_70_. Summing up the “antioxidant part”, we can say that C_60_ and Gd@C_82_ were more effective inducers of the anti-inflammatory pathway than C_70_.

Limitations. Here, we have studied the effects of aqueous fullerene dispersions on human fetal lung fibroblasts. These cells are an excellent model for studying general cell properties. We understand that studies on several lines are needed for more substantiated conclusions. Therefore, we will continue studying the effects of AFDs in mesenchymal stem cells and cancer cells.

## 4. Materials and Methods

### 4.1. AFD Preparation and Characterization

#### 4.1.1. Reagents

Pristine C_60_ and C_70_ (>99.5%) fullerenes were purchased from Limited Liability Scientific and Production Company NeoTechProduct (Saint Petersburg, Russia). The soot containing the Gd@C_2n_ endohedral metal fullerenes (total content of Gd atoms up to 4 wt.% checked by ICP-OES, and the value of total Gd was recalculated to the general formula of the molecule), Gd@C_82_ has been synthesized by the evaporation of the composite graphite electrodes compounded by gadolinium in the electric arc reactor as we previously described elsewhere [34]. Standard reference materials and quality control standards of required elements with certified values (Inorganic Ventures, Christiansburg, VA, USA) were used to conduct ICP-OES measurements. A 20 ppm (in 5 wt.% HNO_3_) scandium solution was used as an internal standard. Ultrapure water Milli-Q^®^ Type (Merck, Germany) was applied during the research (total organic carbon < 3 ppb).

#### 4.1.2. Preparation of Aqueous Fullerene Dispersions by Direct Ultrasound Probe Sonication

AFDs were prepared by the direct sonication with a commercially available off-the-shelf ultrasound probe with a timer MEF93.T (LLC MELFIZ-ul’trazvuk, Moscow, Russia). Ultrasonic tip (surface areas 0.63 ± 0.02 cm^2^) and electrical-power mode (0.6 kW) were used. Ultrasound tips were made of titanium alloys, grade TM3 (ISO 28401:2010). The weighted fullerene portion of ca. 0.05 g, 10 mL of toluene, and 50 mL of ultrapure water were subsequently added to a conical flask (250 mL). The solution was exposed to ultrasonic treatment for 12 h with a pause for 30 min every 60 min. The prepared solution was filtered through a 0.45 μm cellulose filter and diluted to the mark with 50 mL of ultrapure water.

#### 4.1.3. Characterization

Particle size distribution, electrokinetic potential ζ, and polydispersity index were determined according to ISO 22412:2017. The results were verified using two analyzers under the same conditions. A Brookhaven Omni (Brookhaven National Lab, Upton, NY, USA) and a Malvern Zetasizer Nano ZS (Malvern Panalytical, Great Malvern, UK) were used in the 5–1000 nm size range. Measurement angles were 90°, 15°, 173° for Brookhaven Omni and 175° for Malvern Zetasizer Nano ZS.

An Agilent 720 ICP-OES spectrometer (Agilent, Santa Clara, CA, USA) with an axial view was used for elemental analysis.

MALDI MS spectra were recorded using AutoFlex II MALDI TOF/TOF mass spectrometer (Bruker Daltonics, Billerica, MA, USA). MALDI-ToF spectra were acquired by 50 shots in the positive and negative ion reflector mode with a 337-nm molecular nitrogen laser at 80% its power within a mass range from 400 to 1600–5000 Da. α-Cyano-4-hydroxycinnamic acid in a mixture of 30:70 (*v*/*v*) acetonitrile: 0.1% trifluoroacetic acid in water was used as a matrix on an MTP 384 ground steel plate.

The analysis by IR spectroscopy was carried out on a Vertex 70 IR Fourier spectrometer (Bruker Optik GmbH, Ettingen, Germany) with a GladiATR attachment for a single attenuated total internal reflection (ATR) with a diamond crystal (Pike Technologies, Madison, WI, USA).

As we showed previously, spectrophotometry has proved itself well for estimating the concentration of fullerenes in aqueous dispersions [57]. UV/visible absorption spectra were recorded using a two-beam spectrophotometer Cary 4000 (Agilent, Santa Clara, CA, USA). Before the spectrophotometric measurements, we assessed the total non-volatile organic carbon by total organic carbon analysis, as well as other organic components using HPLC-UV or GC-MS. After the total concentration had been determined, calibration UV/visible spectra were recorded, the apparent molar absorptivities (ε) were calculated to estimate fullerene concentrations. This technique works well for conventional C_60_ and C_70_ fullerenes [56]. For endohedral fullerenes, we used the most express approach based on direct measurement of gadolinium in the sample by ICP-OES.

High-resolution images of the aqueous dispersions of C_60_, C_70_, and Gd@C_82_ were obtained using a JEM 2100F field emission electron microscope (Jeol, Tokyo, Japan) with an accelerating voltage of 200 kV. For transmission electron microscopy, the aqueous fullerene dispersions were concentrated up to 200 times by ultracentrifugation at 30 krpm for 45 min at 4 °C. To increase the volatility of the solvent, 200 μL of ethanol was added to 1.00 mL of the concentrated AFD solutions [57].

### 4.2. Cell Culture

The Research Centre for Medical Genetics (RCMG) provided human fetal lung fibroblasts (HFLF) (the 2nd–6th cell passage). Approval#5 was obtained from the Committee for Medical and Health Research Ethics of RCMG. Cells were seeded at 1.7 × 10^4^ per mL in DMEM (Paneco, Moscow, Russia) with a 10% fetal calf serum (PAA Laboratories, Vienna, Austria), 50 U/mL penicillin, 50-μg/mL streptomycin, and 10-μg/mL gentamycin (all the reagents were from Sigma-Aldrich, St. Louis, MO, USA) and cultured at 37 °C for 2 or 24 h, as described elsewhere [19,20]. Various concentrations of AFDs were added to the cells. The cells were incubated for time intervals ranging from 1 h to 72 h.

Two cultures of the same line of HFLF were used. The data obtained were completely consistent with each other. The article presents the total results for two cultures.

### 4.3. MTT Assay and Mitotracker Test

Cell viability was assessed with the 3-(4,5-dimethylthiazol-2-yl)-2,5-diphenyltetrazolium bromide (MTT) assay, as described previously [19,20]. Cells were incubated with AFDs in a 96-well plate for 72 h. The plates were read at 550 nm with EnSpire plate reader (EnSpire Equipment, Turku, Finland). MTT was purchased from Sigma-Aldrich, St. Louis, MO, USA.

The mitotracker test was carried out using a membrane-voltage-dependent dye, tetramethylrhodamine methyl ester (TMRM) (Thermo Fisher, Waltham, MA, USA). TMRM is a cell-permeant, cationic, red-orange fluorescent dye that is readily sequestered by active mitochondria.

### 4.4. Reactive Oxygen Species Assays

Protein expression was assessed with flow cytometry using specific antibodies on a flow cytometer CyFlow Space (Partec, Meckenheim, Germany). Cells were washed with a Versene solution (Thermo Fisher Scientific, Waltham, MA, USA), treated with 0.25% trypsin (Paneco, Moscow, Russia), washed with the culture medium, and suspended in phosphate buffer solution (pH 7.4) (Paneco, Moscow, Russia). The cells were fixed with paraformaldehyde (PFA, Sigma-Aldrich, Saint Louis, MO, USA) at 37 C for 10 min, then washed three times with 0.5% BSA–PBS and permeabilized with 0.1% Triton X-100 (BSA and Triton X-100 were from Sigma-Aldrich, Saint Louis, MO, USA) in PBS for 15 min at 20 °C or with 90% methanol (Sigma-Aldrich, Saint Louis, MO, USA) at 4 °C, then washed with 0.5% BSA–PBS (3 times). The cells were stained with conjugated antibodies (1 µg/mL) for 2 h at room temperature, washed with PBS, and analyzed by a flow cytometer (CytoFlex S, Beckman Coulter, Brea, CA, USA).

### 4.5. Quantification of mRNA Levels

Gene expression was assessed by real-time polymerase chain reaction (PCR). After exposure to fullerenes, RNA was isolated from the cells using YellowSolve kits (Klonogen, St. Petersburg, Russia) according to the standard procedure, followed by phenol–chloroform extraction and precipitation with chloroform and isoamyl alcohol (49:1). The RNA concentration was determined using the Quant-iT RiboGreen RNA reagent (MoBiTec, Göttingen, Germany) on a plate reader (EnSpire equipment, Turku, Finland), λ_ex_ = 487 nm, λ_fl_ = 524 nm. According to the standard procedure, the reverse transcription reaction was carried out using reagents from Sileks (Moscow, Russia). PCR was performed using the appropriate primers (Synthol) and the SYBR Green PCR Master Mix (Applied Biosystems, Foster City, CA, USA) on a StepOnePlus device (Applied Biosystems, Foster City, CA, USA). The technical error was approximately 2%. TBP was used as a reference gene.

### 4.6. Fluorescence Microscopy

An Axio Scope.A1 microscope (Carl Zeiss, Oberkochen, Germany) wase used for fluorescent microscopy of cells. The fluorescence experiments were performed in a 6-well plate at a cell concentration of 10^6^ cells/well. No less than 100 fields of view were analyzed; fluorescence intensity per a cell and the total fluorescence were analyzed using microscope software.

### 4.7. Statistical Analysis

Experiments were repeated in triplicate. In FCA, the medians of the signal intensities were analyzed. Figures show the mean and standard deviation (SD). The significance of the observed differences was analyzed with the nonparametric Mann–Whitney *U*-test. The *p*-values < 0.01 were considered statistically significant and marked on figures with the “∗” sign. The data were analyzed with Excel, Microsoft Office (Microsoft, Redmond, USA), Statistica 6.0 (Dell Round Rock, TX, USA), and StatGraphics (Statgraphics Technologies, The Plains, VA, USA).

## 5. Conclusions

The aqueous dispersions of C_60_, C_70_, and Gd@C_82_ fullerenes are active participants in ROS homeostasis. Low and high concentrations of AFDs have similar effects, and we can hypothesize that these effects are monotonic within the cell viability range. The studied effects were various in strength. C_70_ was the most inert substance, C_60_ was the most active substance. They all have both a “prooxidant” and “antioxidant” effect, but with a different balance. Presumably, Gd@C_82_ was a substance with more pronounced antioxidant and anti-inflammatory properties, while C_70_, on the contrary, had more pronounced “prooxidant” properties. A more comprehensive understanding of the role of fullerenes will be obtained after studying their effect on DNA damage and the adaptive response (activation of repair systems, autophagy, and apoptosis), which is the aim of our subsequent studies.

## Figures and Tables

**Table 1:** Concentrations (*c*), size, zeta-potentials (ζ), and polydispersity indices (PDI) of the AFDs.

Fullerene	c, mM	Particle Size after a 0.22 μm Filter, nm	ζ-potential, mV	PDI
C_60_	0.083 ^1^	110 ± 5	−28.4 ± 0.2	0.109 ± 0.003
C_70_	0.081 ^1^	113 ± 2	−29.5 ± 0.3	0.160 ± 0.008
Gd@C_82_	0.022 ^2^	95 ± 5	−32.3 ± 0.3	0.123 ± 0.008

^1^ Measured by UV/vis spectroscopy. ^2^ Measured by ICP–OES.

## Data Availability

The datasets used and/or analyzed during the current study are available from the corresponding author on reasonable request.

## Supplementary Materials

The following are available online at https://www.mdpi.com/article/10.3390/ijms22116130/s1.
