# Supplementary material for: Effects of Aqueous Dispersions of C60, C70 and Gd@C82 Fullerenes on Genes Involved in Oxidative Stress and Anti-Inflammatory Pathways
Source: Int J Mol Sci. 2021 Jun 7;22(11):6130. doi: 10.3390/ijms22116130 (PMC8201376; doi:10.3390/ijms22116130)
Supplement: Supplementary file 1 [file ijms-22-06130-s001.zip › ijms-1227135-supplementary.pdf]

International Journal of
Molecular Sciences

MDPI

Supplementary Materials

Effects of Aqueous Dispersions of C_{60}, C_{70} and Gd@C_{82} Fullerenes on Genes Involved in Oxidative Stress and Anti-inflammatory Pathways: Supplementary Materials

Elena V. Proskurnina, Ivan V. Mikheev, Ekaterina A. Savinova, Elizaveta S. Ershova, Natalia N. Veiko, Larisa V. Kameneva, Olga A. Dolgikh, Ivan V. Rodionov, Mikhail A. Proskurnin, and Svetlana V. Kostyuk

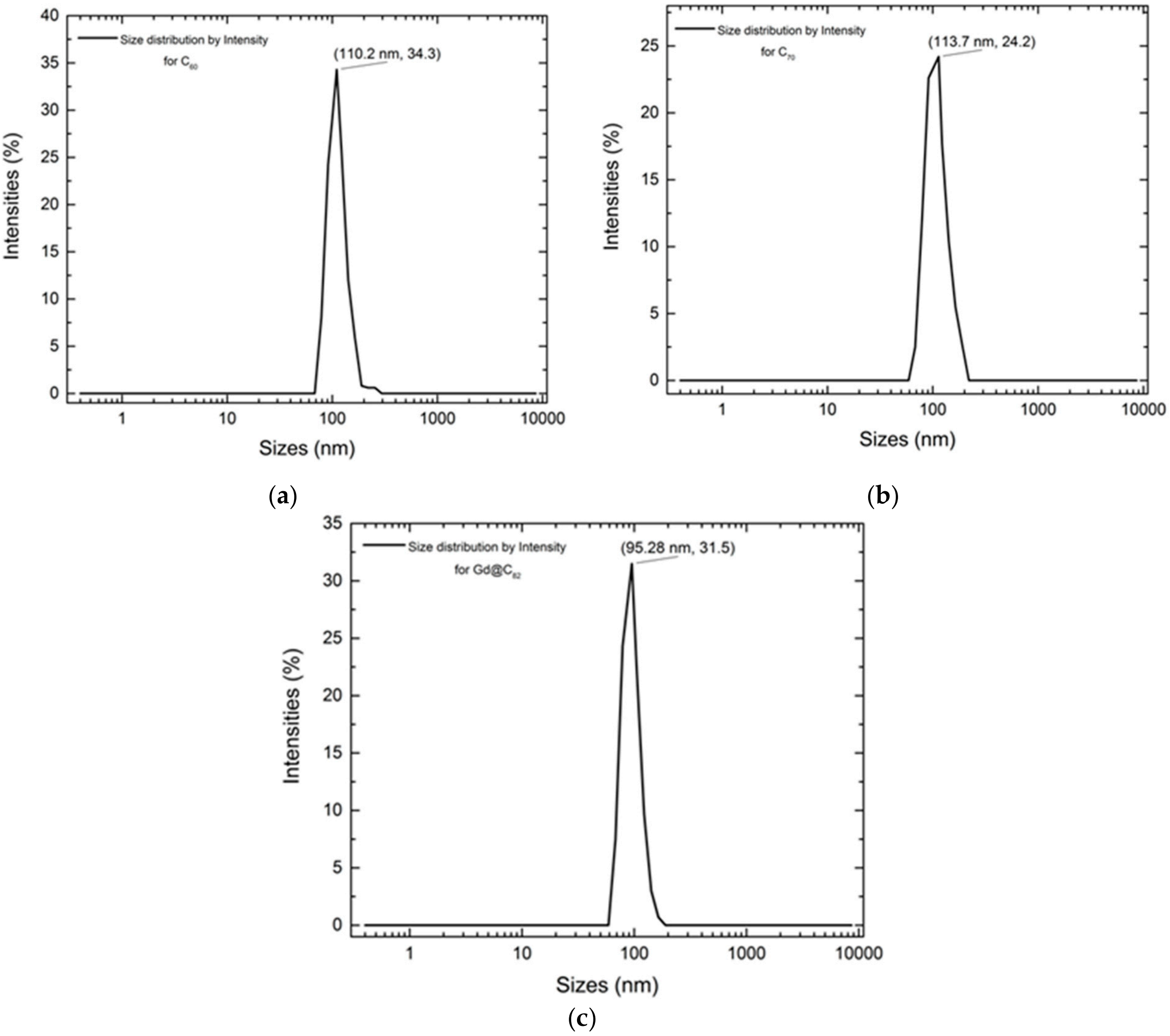

Figure S1. Dynamic light scattering profiles for C_{60} (**a**), C_{70} (**b**), and Gd@C_{82} (**c**).

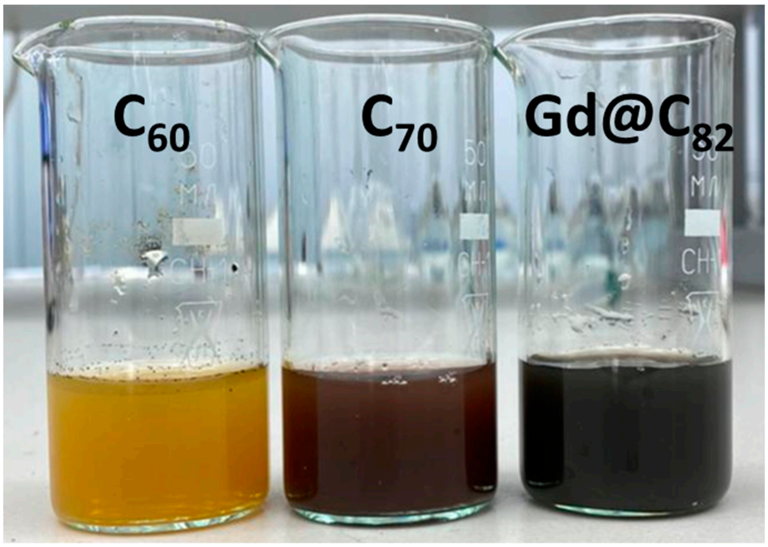

Figure S2. Photographs of the aqueous dispersions of C_{60}, C_{70}, and Gd@C_{82}.

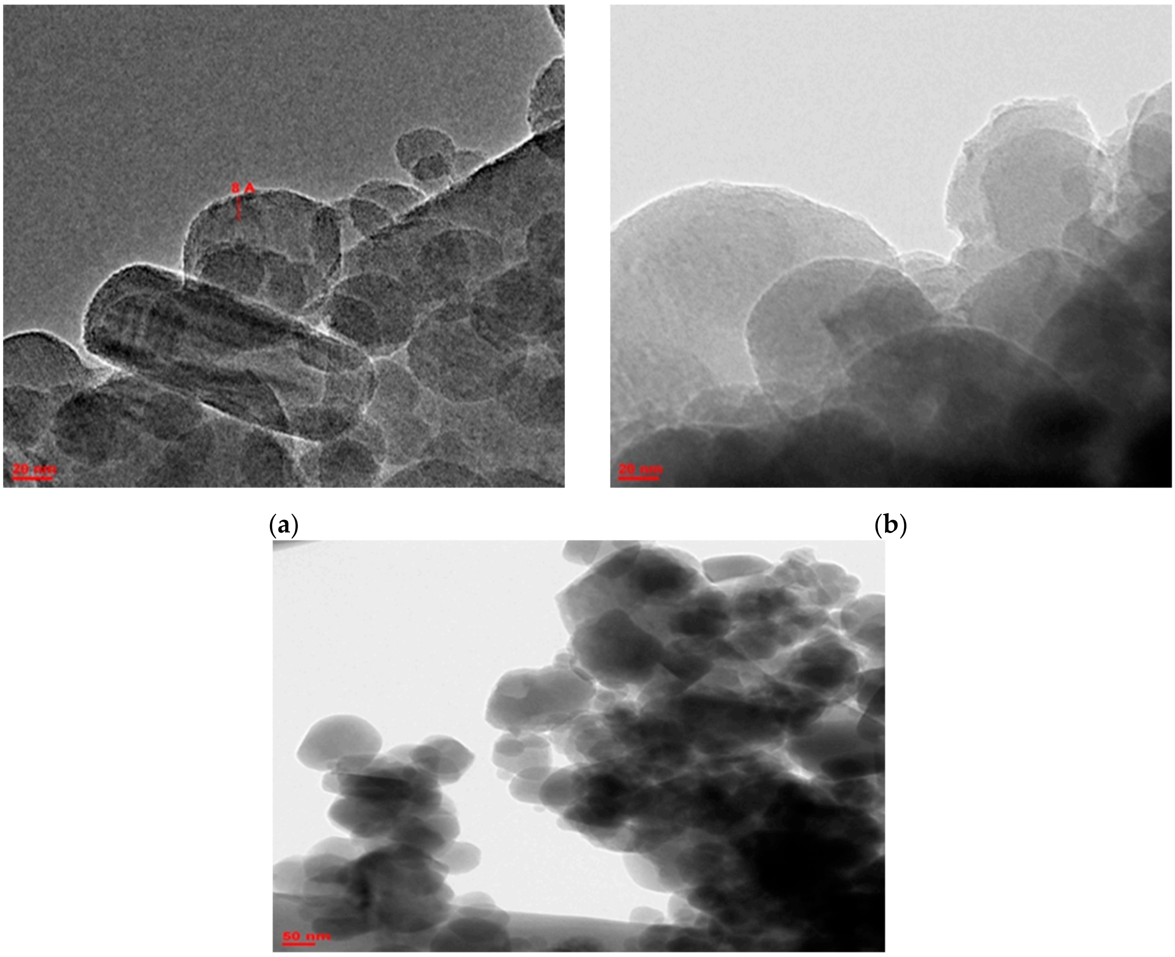

(**a**) (**b**)

(**c**)

Figure S3. TEM images for C_{60} (**a**), C_{70} (**b**), and Gd@C_{82} (**c**) obtained with a JEM 2100F microscope, accelerating voltage was 200 kV; scale in the picture for (a) and (c) is 20 nm, for (b) is 50 nm [Mikheev, I.V.; Usoltseva, L.O.; Ivshukov, D.A.; Volkov, D.S.; Korobov, M.V.; Proskurnin, M.A. Approach to the assessment of size-dependent thermal properties of disperse solutions: Time-resolved photothermal lensing of aqueous pristine fullerenes C_{60} and C_{70}, *J. Phys. Chem. C*, **2016**, *120*, 28270–28287, doi:10.1021/acs.jpcc.6b08862].

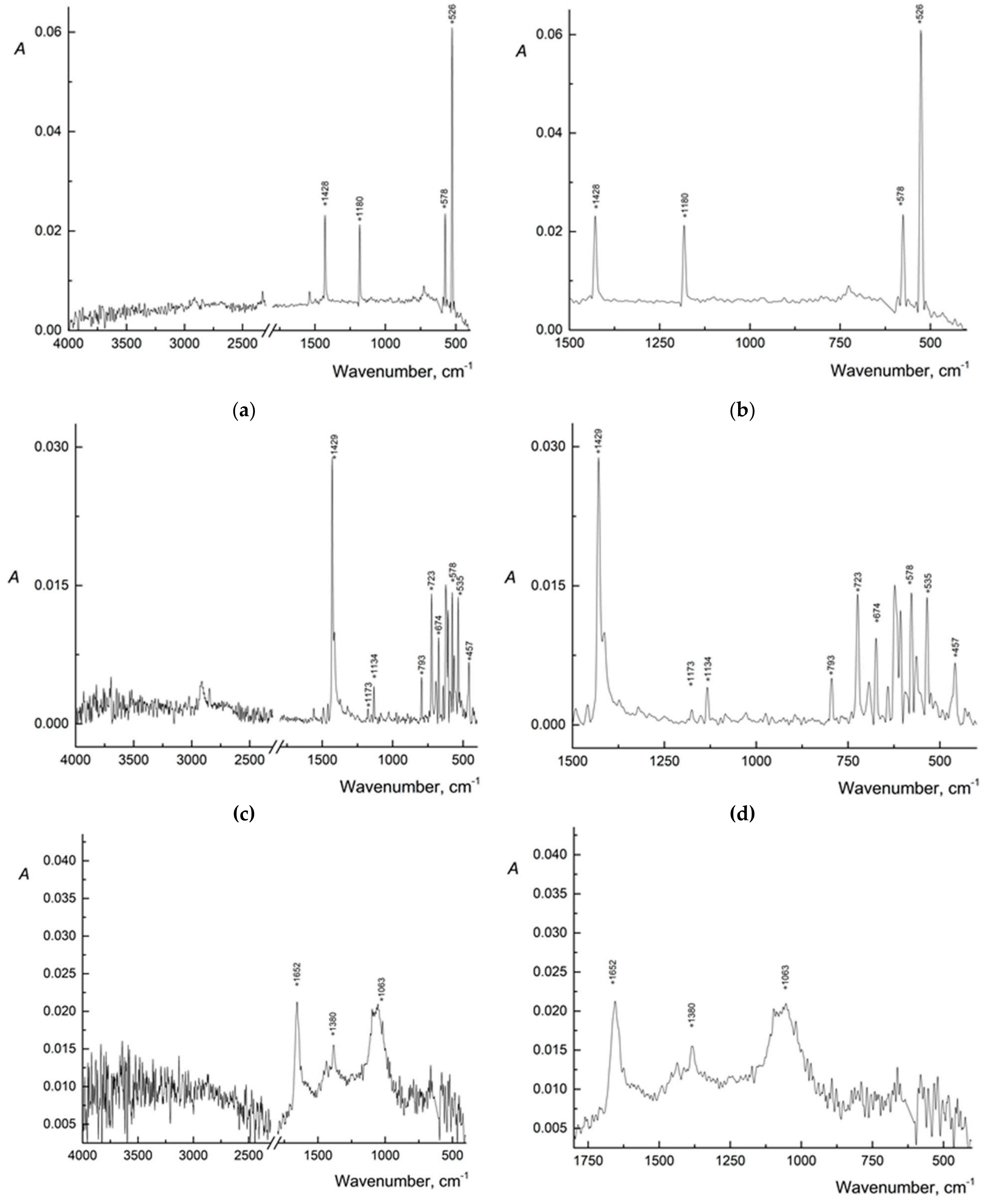

Figure S4. Fourier-transform infrared spectra registered with Bruker Vertex 70 (**a, c, e**) and with a GladiATR attachment for a single attenuated total internal reflection with a diamond crystal (**b, d, f**) of aqueous dispersions of C_{60} (a, b), C_{70} (c, d), and Gd@C_{82} (e, f).
